# Mouse and Human CD1d-Self-Lipid Complexes Are Recognized Differently by Murine Invariant Natural Killer T Cell Receptors

**DOI:** 10.1371/journal.pone.0156114

**Published:** 2016-05-23

**Authors:** Tingxi Guo, Kenji Chamoto, Munehide Nakatsugawa, Toshiki Ochi, Yuki Yamashita, Mark Anczurowski, Marcus O. Butler, Naoto Hirano

**Affiliations:** 1 Tumor Immunotherapy Program, Campbell Family Institute for Breast Cancer Research, Campbell Family Cancer Research Institute, Princess Margaret Cancer Centre, University Health Network, Toronto, Ontario, Canada; 2 Department of Immunology, University of Toronto, Toronto, Ontario, Canada; 3 Department of Medicine, University of Toronto, Toronto, Ontario, Canada; University Paris Sud, FRANCE

## Abstract

Invariant natural killer T (iNKT) cells recognize self-lipids presented by CD1d through characteristic TCRs, which mainly consist of the invariant Vα14-Jα18 TCRα chain and Vβ8.2, 7 or 2 TCRβ chains with hypervariable CDR3β sequences in mice. The iNKT cell-CD1d axis is conserved between humans and mice, and human CD1d reactivity of murine iNKT cells have been described. However, the detailed differences between the recognition of human and mouse CD1d bound to various self-lipids by mouse iNKT TCRs are largely unknown. In this study, we generated a *de novo* murine iNKT TCR repertoire with a wider range of autoreactivity compared with that of naturally occurring peripheral iNKT TCRs. Vβ8.2 mouse iNKT TCRs capable of recognizing the human CD1d-self-lipid tetramer were identified, although such clones were not detectable in the Vβ7 or Vβ2 iNKT TCR repertoire. In line with previously reports, clonotypic Vβ8.2 iNKT TCRs with unique CDR3β loops did not discriminate among lipids presented by mouse CD1d. Unexpectedly, however, these iNKT TCRs showed greater ligand selectivity toward human CD1d presenting the same lipids. Our findings demonstrated that the recognition of mouse and human CD1d-self-lipid complexes by murine iNKT TCRs is not conserved, thereby further elucidating the differences between cognate and cross-species reactivity of self-antigens by mouse iNKT TCRs.

## Introduction

Invariant natural killer T (iNKT) cells are lipid reactive T cells restricted by the monomorphic MHC class I homolog CD1d. This subset of T cells is characterized by the recognition of the glycolipid α-galactosylceramide (α-GalCer) or its analog PBS-57. They rapidly respond to activation by secreting cytokines which is characteristic of both Th1 and Th2 responses, and they have been implicated to play a role in a variety of diseases. The TCRs of iNKT cells are more restricted compared with conventional T cells, with the TCRα chain largely limited to Vα14-Jα18 in mice and Vα24-Jα18 in humans. TCRβ usage is biased toward Vβ8.2, 7, and 2 in mice and Vβ11 in humans with hypervariable CDR3β sequences [1–6].

Previous reports have demonstrated the importance of self-lipids presented by CD1d in iNKT cell biology. The recognition of self-lipids is crucial in the development, maintenance, and activation of iNKT cells [7–11]. Some of the previously identified self-ligands include lyso-phosphatidylcholine (LPC) [12, 13]; β-glucopyranosylceramide (β-GlcCer), whose antigenicity in commercial preparations is most likely due to contaminating α-GlcCer [14–16]; C16-lysophosphatidylethanolamine (pLPE); and C16-alkanyl-lysophosphatidic acid (eLPA) [17]. Structural studies have elucidated the molecular basis of the autoreactivity of iNKT TCR, and have found that the TCRα chain dominates the interaction, in a manner similar to that in the recognition of α-GalCer-CD1d by iNKT TCRs. The key difference between the recognition of self-lipids and the canonical lipid is in the role of CDR3β. This loop negligibly contributes to the recognition of α-GalCer-CD1d, but it mediates direct contact with CD1d presenting self-lipids [2, 12, 18–22].

In cognate systems, it has been shown that clonotypic iNKT TCRs are unable to preferentially recognize different CD1d-self-lipid complexes, unlike how different MHC-restricted TCRs are able to selectively recognize antigens [20, 21, 23]. This is consistent with their respective structural data, which have shown that the diverse CDR3 loops of conventional TCRs directly interact with the peptide, whereas the CDR3β loop of iNKT TCRs only interacts with the monomorphic antigen-presenting molecule [2]. In other words, the hierarchy of the reactivity of iNKT TCRs is conserved, regardless of the lipid presented by CD1d. This phenomenon limits the possibility of CD1d-lipid specific iNKT cell-based immunotherapy, since high affinity iNKT TCRs can target virtually all CD1d-restricted antigens without discriminating the antigen of interest from other antigens.

Interestingly, murine iNKT cells can also recognize human CD1d (hCD1d)-lipid complexes [24]. This process is largely mediated by the homology of murine Vα14 and human Vα24, murine Vβ8.2 and human Vβ11, and the CD1d genes [25, 26]. However, a comprehensive analysis of murine iNKT TCR cross-species recognition at the repertoire and clonal level is lacking, especially for CD1d presenting self-lipids. Here, we have generated a *de novo* repertoire of murine iNKT TCRs, which we studied at both the population and the clonotypic levels. Using human and mouse CD1d (mCD1d) tetramers loaded with self-lipids, we found that while Vβ8.2, 7, and 2 iNKT TCR repertoires all included self-lipid mCD1d tetramer positive clones, only the Vβ8.2 repertoire possessed clones capable of recognizing self-lipid hCD1d tetramer. Importantly, all clonotypic Vβ8.2 iNKT TCRs tested recognized mCD1d presenting various self-lipids in a conserved hierarchy. However, they recognized hCD1d that presented the same lipids with significantly more ligand selectivity. These data support the potential use of hCD1d-lipid specific immunotherapy using mouse iNKT TCRs [27].

## Materials and Methods

### Mice, Cells, and Reagents

C57BL/6 mice and CD1d^-/-^ (C57BL/6 background) mice were purchased from the Jackson Laboratory (Bar Harbor, ME). All animal studies were approved by the Animal Research Core of the University Health Network (Protocol ##2462.8 and #2623.6), and performed in accordance with the guidelines of Canadian Council on Animal Care. Euthanasia was performed using carbon dioxide. EL4 cells (ATCC, Manassas, VA), a thymocyte cell line from the C57BL/6 background, were cultured in DMEM supplemented with 10% fetal calf serum (FCS) and gentamicin (Life Technologies, Carlsbad, CA). Primary mouse T cells were cultured in RPMI 1640 supplemented with 10% FCS, gentamicin, 2-mercaptoethanol, HEPES, and sodium pyruvate (Sigma-Aldrich, St. Louis, MO). 5KC cells were cultured in RPMI supplemented with 10% FCS, gentamicin, 2-mercaptoethanol, HEPES, and sodium pyruvate, and non-essential amino acids (Wisent Bioproducts, St. Bruno, Quebec, Canada). 5KC cells were originally described in White et al [28]. Jurkat 76 cells and their derivatives were cultured in RPMI 1640 supplemented with 10% FCS and gentamicin. Jurkat 76 cells were originally described in Heemskerk et al [29]. α-Galactosylceramide (α-GalCer) was purchased from Axxora (San Diego, CA), and β-glucopyranosylceramide C_24:1_ (β-GlcCer), lyso-phosphatidylcholine (18:1) (LPC), C16-alkanyl-lysophosphatidic acid (eLPA) and C16-lysophosphatidylethanolamine (pLPE) were purchased from Avanti Polar Lipids (Alabaster, AL).

### cDNAs

Full-length cDNAs encoding the invariant Vα14 TCRα and Vβ8.2, 7, and 2 TCRβ genes were cloned via RT-PCR using respective V gene and constant region specific primers. The cDNAs were cloned into the pMPSV vector, a derivative of the pJP1520 vector that contains myeloproliferative sarcoma virus long terminal repeats. The sequence information for the pMPSV vector is available upon request. Nucleotide sequencing was performed at the Centre for Applied Genomics, The Hospital for Sick Children (Toronto, Canada).

### Transduction and Expansion of Primary Mouse T Cells

Primary murine splenocytes were stimulated with concanavalin A (Sigma-Aldrich) for 24 hours and were retrovirally transduced with Vα14i-ΔNGFR gene using Phoenix-Eco packaging cells in Retronectin-coated plates (Takara Bio, Japan). The cells were cultured in the presence of 100 IU/ml of recombinant human IL-2 (Novartis, New York, NY). Artificial APCs were irradiated (100 Gy) and added to the responder cells at a responder to stimulator ratio of 10:1 after infection. The T cells were restimulated every 6 days for a total of two stimulations and supplemented with 20 IU/ml of IL-2 and 20 ng/ml of IL-15 (Peprotech, Rocky Hill, NJ) every three to four days. The transduction efficiency ranged from 50% to 80% of the total T cells, as determined by anti-NGFR mAb staining, and the expression was maintained for at least 3 weeks post transduction.

### Generation of CD1d-Artificial Antigen-Presenting Cells (aAPC)

EL4-based CD1d-artificial antigen-presenting cells (aAPC) were generated by retrovirally transducing mouse CD80 into EL4 cells using 293GPG packaging cells as previously published [30–33]. CD80-positive cells were isolated by using MACS Cell Separation (Miltenyi Biotec, Germany) following mAb staining.

### Flow Cytometry Analysis

Monoclonal antibodies (mAbs) that recognize the following antigens were used: mouse CD3 (clone 17A2), CD80 (16-10A1), CD1d (clone K253); human CD3 (clone UCHT1), CD4 (clone RPA-T4), CD8α (RPA-T8), CD1d (clone 51.1), CD69 (clone FN50), CD271 (NGFR, clone ME20.4), and isotype controls from BioLegend (San Diego, CA). Human and mouse CD1d tetramers, both unloaded and loaded with the α-GalCer analog PBS-57, were kindly provided by the NIH Tetramer Core Facility. The unloaded monomers were produced in HEK293 cells and therefore presented HEK293-derived endogenous ligand(s). Where indicated, the unloaded tetramers were loaded with β-GlcCer, LPC, pLPE, or eLPA according to the protocol provided by the Tetramer Core Facility. All PBS-57 loaded tetramers were stained at 1 μg/ml. The unloaded, β-GlcCer, pLPE, and eLPA loaded tetramers were used at 10 μg/ml. The surface molecule staining and subsequent flow cytometry analysis were performed as described elsewhere [34]. All data were gated on total live cells, except for when analyzing 5KC transfectants expressing clonotypic TCRs, where live and the CD3^+^ cells were gated.

### Generation of TCR transfectants

The Vα14i TCRα gene tagged with ΔNGFR was transduced into TCR^-/-^ 5KC cells. The clone Jurkat 76.3E1 was established by serially infecting Jurkat 76 cells with the CD4, CD8αβ, and Vα14i TCRα-ΔNGFR genes as previously reported (35–37). Surface CD1d expression was knocked down with the transduction of a retroviral vector encoding a β2-microglobulin-targeting shRNA (OriGene Technologies, Rockville, MD). 5KC-expressing Vα14i TCRα and 3E1 cells were then reconstituted with the TCRβ genes. All retroviral infection of the T cell lines used 293GPG. All Jurkat 76.3E1 transfectants had >90% CD3 positivity. HLA-A2/TAX TCR (A6) was used as the control TCR [35].

### CD69 Upregulation Assay

Human or mouse CD1d monomers (5 μg/mL, NIH tetramer core facility) were coated onto 96-well ELISA plates (Thermo Scientific, Waltham, MA) along with anti-human CD28 mAb (2 μg/mL, BioLegend) for 2 hours at 37°C. Lipids (10 μg/mL) were then added and incubated for 16 hrs at 37°C. Twenty five thousand Jurkat 76.3E1 transfectant cells were added to each well and cultured for 6 hours. The upregulation of CD69 surface expression was analyzed by flow cytometry.

### Statistical Analysis

Statistical analysis was performed using GraphPad Prism 6.0. The non-parametric Spearman’s rank correlation coefficient values greater than 0.7 were considered highly correlated, values in the range between 0.4 and 0.7 moderately correlated, and values less than 0.4 weakly correlated. All statistical tests were two-sided, and a *p* value of <0.05 was considered significant.

## Results

### Cloning of Mouse iNKT TCRs with High Autoreactivity

Despite their autoreactivity, peripheral iNKT cells experience thymic selection, which imposes a limit on the reactivity range of naturally occurring iNKT TCRs in the periphery against self-ligands [8]. Therefore, to better model how mouse iNKT TCRs recognize self-ligands, it is necessary to generate an iNKT TCR repertoire that includes TCRs possessing autoreactivity beyond the limit of CD1d-restricted thymic selection. It has been reported that iNKT cells and conventional MHC-restricted T cells both develop from double positive thymocytes [36]. Because of this, their TCRβ chain repertoires partially overlap. Therefore, by pairing the peripheral TCRβ chain repertoire of MHC-restricted TCRs with the invariant Vα14 (Vα14i) TCRα chain, we should be able to retrieve at least a subset of mouse iNKT TCRs with high autoreactivity. We have previously generated *de novo* repertories of human iNKT TCR and tumor-reactive HLA-restricted TCR through a similar strategy [37–39].

**Fig 1:**
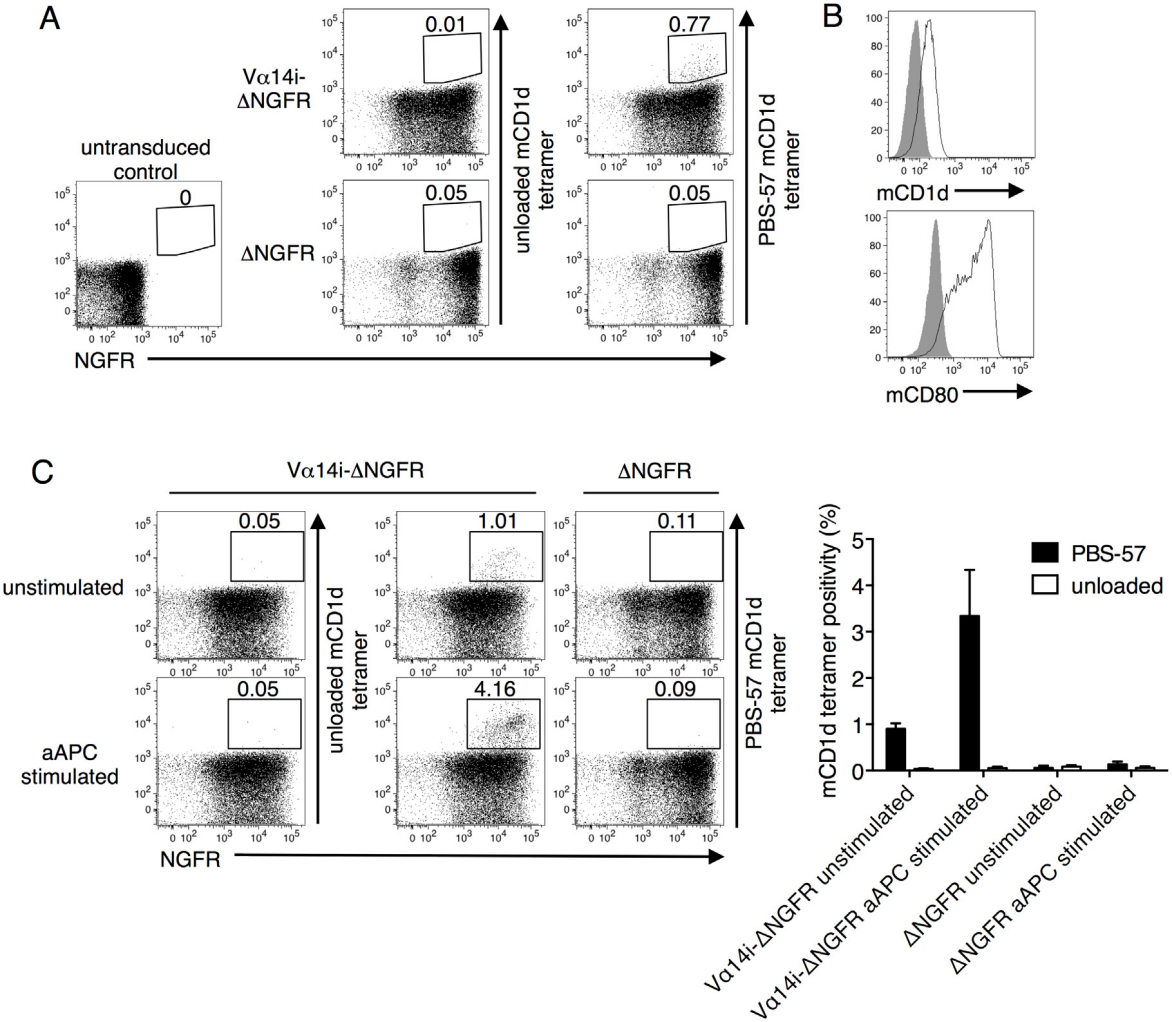
Generating *de novo* mouse iNKT TCRs. (A) Splenocytes from CD1d knockout (KO) mice of the C57BL/6 background were transduced with the full-length invariant Vα14 TCRα (Vα14i) chain, tagged with the extracellular portion of human nerve growth factor receptor (ΔNGFR), or the tag alone. The transfectants were stained with anti-NGFR mAb and unloaded or PBS-57 loaded mouse CD1d (mCD1d) tetramer. (B) EL4-based artificial APCs (aAPC) were stained with anti-mCD1d and anti-mouse CD80 mAbs (black lines). The solid gray color indicates the control. (C) Vα14i-transduced CD1d^-/-^ splenocytes were stimulated with aAPC and then stained with anti-NGFR mAb and unloaded or PBS-57 loaded mCD1d tetramer. Mean percentages ± SD are shown in the graph. The data are representative of three independent experiments.

We cloned the full-length mouse Vα14i gene linked to a truncated form of the nerve growth factor receptor (ΔNGFR) gene as a tag, and retrovirally transduced primary CD1d^-/-^ splenocytes with the construct. CD1d^-/-^ mice lack endogenous iNKT cells. The tag alone was transduced as a control. We observed PBS-57 mouse CD1d (mCD1d) tetramer positivity in cells transduced with Vα14i but not in control transduced cells (Fig 1A). Thus, introducing the Vα14i gene generated *de novo* iNKT TCRs. To selectively enrich for iNKT TCRs with higher self-antigen reactivity, we developed EL4-based artificial antigen presenting cell (aAPC) system. EL4, a syngeneic thymocyte cell line that endogenously expresses CD1d that presents self-ligands, was transduced with CD80 to confer a costimulatory signal (Fig 1B). When stimulated twice with the irradiated and unpulsed aAPCs, the frequency of PBS-57 mCD1d tetramer positivity increased in the Vα14i-transduced CD1d^-/-^ cells but not the control cells (Fig 1C). However, prior to or following aAPC stimulation, the Vα14i-transduced T cells did not possess sufficient avidity to be stained by unloaded mCD1d tetramer, which presented unknown self-lipids derived from HEK293 cells (Fig 1A and 1C).

**Fig 2:**
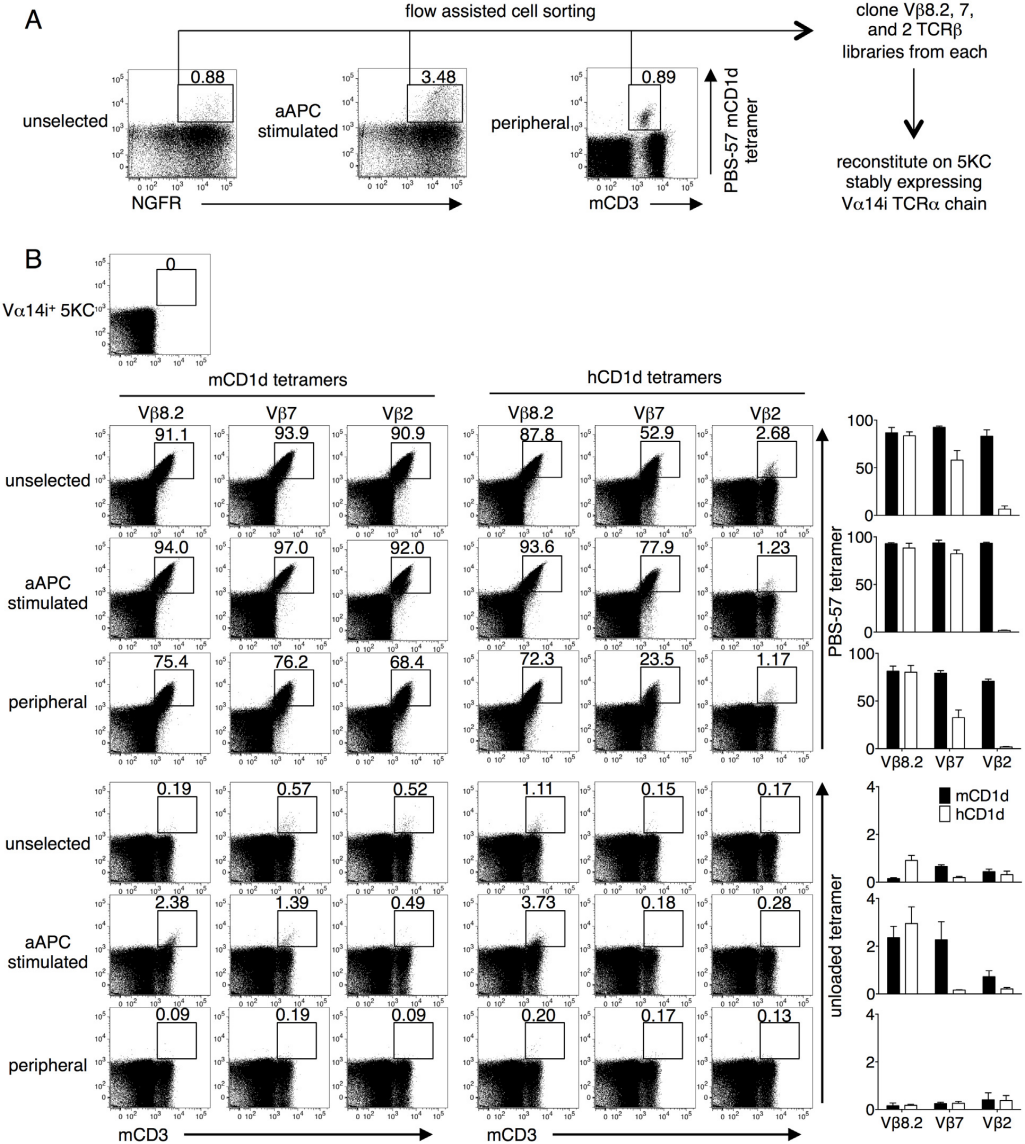
Staining of mouse iNKT TCR library transfectants with mouse and human CD1d tetramers. (A) The total Vβ8.2, 7 and 2 libraries from the PBS-57 tetramer positive populations of Vα14i-transduced CD1d^-/-^ splenocytes (unselected), aAPC-stimulated Vα14i-transduced CD1d^-/-^ splenocytes (aAPC stimulated), and the splenocytes of a wild-type mouse (peripheral) were cloned. Each library was reconstituted in 5KC cells that stably expressed Vα14i. (B) The transfectants were stained with anti-mouse CD3 (mCD3) mAb and stained with the unloaded or PBS-57 loaded mCD1d or hCD1d tetramers. The number above the gate indicates the percentage among CD3^+^ transfectants. Mean percentages ± SD are shown in the graphs. The data are representative of three independent experiments.

We, as well as others, have shown that T cell lines expressing iNKT TCRs display higher structural reactivity, defined as the intensity of CD1d tetramer staining, compared with primary T cells [21, 39]. Using flow cytometry, we collected PBS-57 mCD1d tetramer-positive cells from Vα14i-transduced but unstimulated CD1d^-/-^ T cells (unselected), Vα14i-transduced and subsequently aAPC-stimulated CD1d^-/-^ T cells (aAPC stimulated), and splenocytes of a congenic wild type mouse as a control (peripheral). Total Vβ8.2, 7, and 2 TCRβ genes were amplified and cloned from the three cohorts. Each of the nine libraries (Vβ8.2, 7, and 2 from unselected, aAPC stimulated, and peripheral) was reconstituted in a mouse TCR^-/-^ T cells line, 5KC, that had been stably transduced with Vα14i (Fig 2A). The transduction efficiency was limited to approximately 20% to minimize the frequency of single cells expressing multiple TCRβ chains. Each library was stained with mCD1d or hCD1d, unloaded or PBS-57 tetramer. Transfectants from all the libraries were positively stained for the PBS-57 mCD1d tetramer, indicating that the libraries indeed consisted of mouse iNKT TCRβ chains (Fig 2B, top left).

Importantly, we detected unloaded tetramer-positive cells in unselected and aAPC stimulated library transfectants but not in the transfectants of the peripheral iNKT TCRβ chain libraries (Fig 2B, bottom). In addition, the peripheral libraries were stained with lower intensity with the PBS-57 mCD1d tetramer, suggesting lower structural reactivity. These findings validate our approach in generating a thymically unselected repertoire, followed by stimulating with unloaded CD1d-expressing aAPC and stably reconstituting the TCRs on a TCR^-/-^ T cell line. Interestingly, the Vβ8.2 iNKT TCR library transfectants, but not the Vβ7 or 2 TCR transfectants, were similarly stained with the PBS-57 mCD1d and hCD1d tetramers. In line with this result, the Vβ7 and Vβ2 mouse iNKT TCR transfectants possessed less or nearly lacked hCD1d reactivity, respectively (Fig 2B, top right). Furthermore, while at least some unloaded mCD1d tetramer positivity was observed in all three Vβ repertoires (Fig 2B, bottom left), only Vβ8.2 TCRs were able to recognize the unloaded hCD1d tetramer (Fig 2B, bottom right). Notably, both human and mouse unloaded CD1d tetramers were produced in HEK293 cells. These observations are consistent with the fact that human Vβ11 shows higher sequence homology with mouse Vβ8.2 than with the Vβ7 or Vβ2 genes [25]. Given these findings, Vβ8.2 iNKT TCRs were selected for further analyses.

### Clonotypic Analysis of Mouse iNKT TCRs Recognizing Mouse and Human CD1d

**Fig 3:**
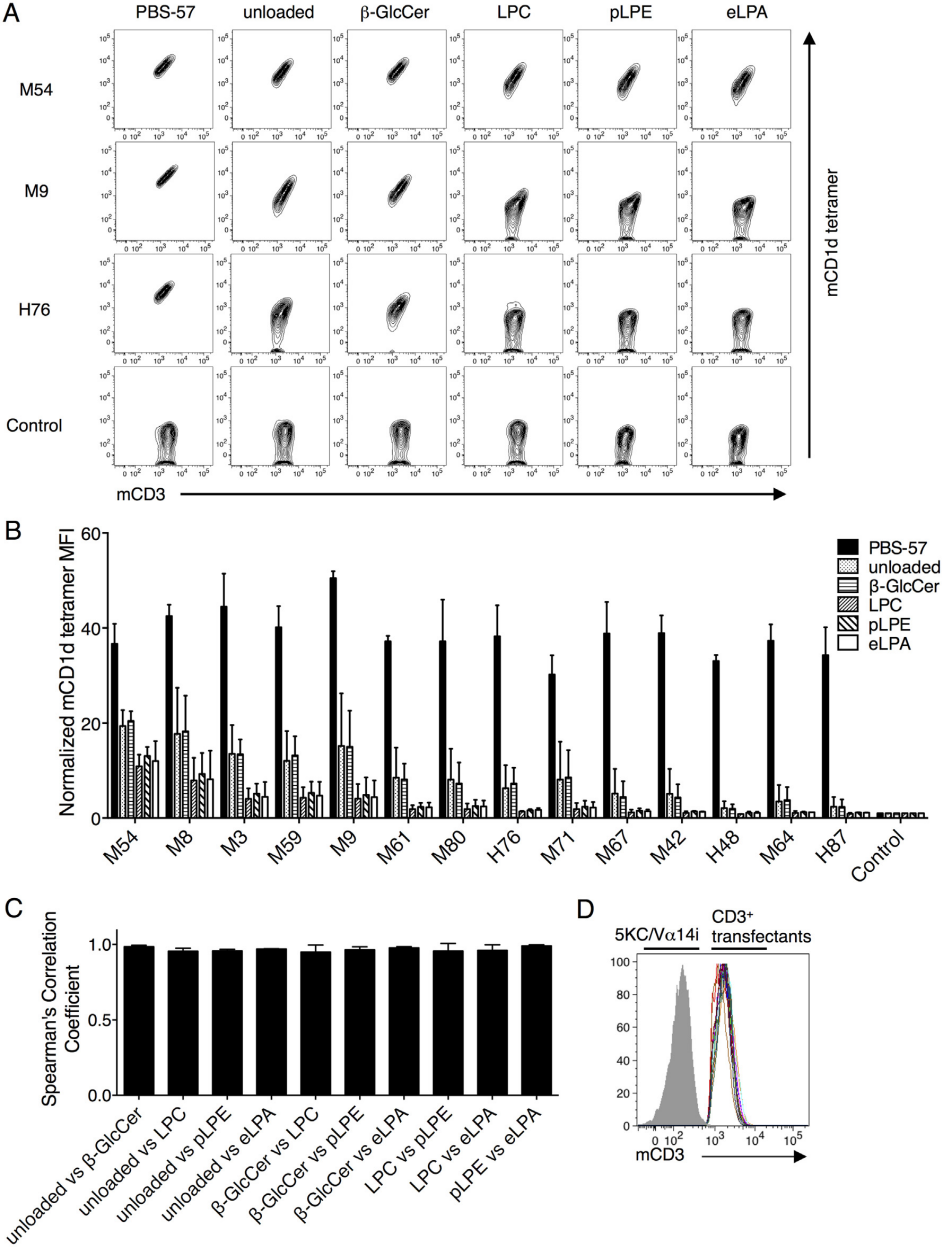
Murine Vβ8.2 iNKT TCRs recognize mCD1d-self-lipid complexes without ligand selectivity. (A and B) Clonotypic Vβ8.2 TCRβ chains were cloned from the unloaded mCD1d and hCD1d tetramer-positive population of the aAPC-stimulated library and reconstituted in 5KC cells expressing Vα14i. Each transfectant was stained with anti-mCD3 mAb and unloaded or PBS-57, β-GlcCer, LPC, pLPE, and eLPA loaded mCD1d tetramers. MFI values shown are based on CD3^+^ cells. The raw data for three representative clones are shown in A. Staining for all other transfectants are shown in S1 Fig. MFI was normalized to that of control transfectants stained with the same tetramer. (C) Spearman correlation coefficients were calculated between the indicated pairs of antigens. (D) CD3 expression of CD3^+^ 5KC transfectants are shown by overlaid multicolor histograms. The gray solid indicates baseline fluorescence of TCR^-/-^ 5KC. Mean values ± SD are shown in the graphs. Data are representative of two independent experiments.

Next, we studied the recognition of self-ligands by mouse iNKT TCRs at the clonal level. Unloaded mCD1d and hCD1d tetramer-positive populations were collected from the aAPC-stimulated Vβ8.2 library, which possessed the highest percentage of unloaded tetramer positivity (Fig 2B, bottom). The TCRs were then cloned and individually reconstituted in the Vα14i-expressing 5KC cells. Each transfectant was stained with mCD1d tetramer, which was unloaded or loaded with PBS-57, β-GlcCer, LPC, pLPE, or eLPA (Fig 3A, 3B and S1 Fig). PBS-57 mCD1d tetramer staining served as a positive control. The tetramer staining of an HLA-restricted TAX TCR (control) 5KC transfectant served as a negative control. The sequence information for the self-lipid mCD1d tetramer-positive Vβ8.2 TCRβ chains is shown in S1 Table.

The MFIs obtained by staining with the self-lipid mCD1d tetramers were highly correlated regardless of the pair analyzed, demonstrating a ligand-nonselective mode of recognition by these iNKT TCRs (Fig 3C). In other words, the sequence differences between the tested iNKT TCRs did not influence the ligand selectivity and only affected the overall affinity for the mCD1d-self-lipid complex. These observations are consistent with previous findings by other investigators [20, 23]. Note that all of the CD3^+^ 5KC transfectants expressed comparable levels of CD3 (Fig 3D), therefore can be directly compared to each other.

**Fig 4:**
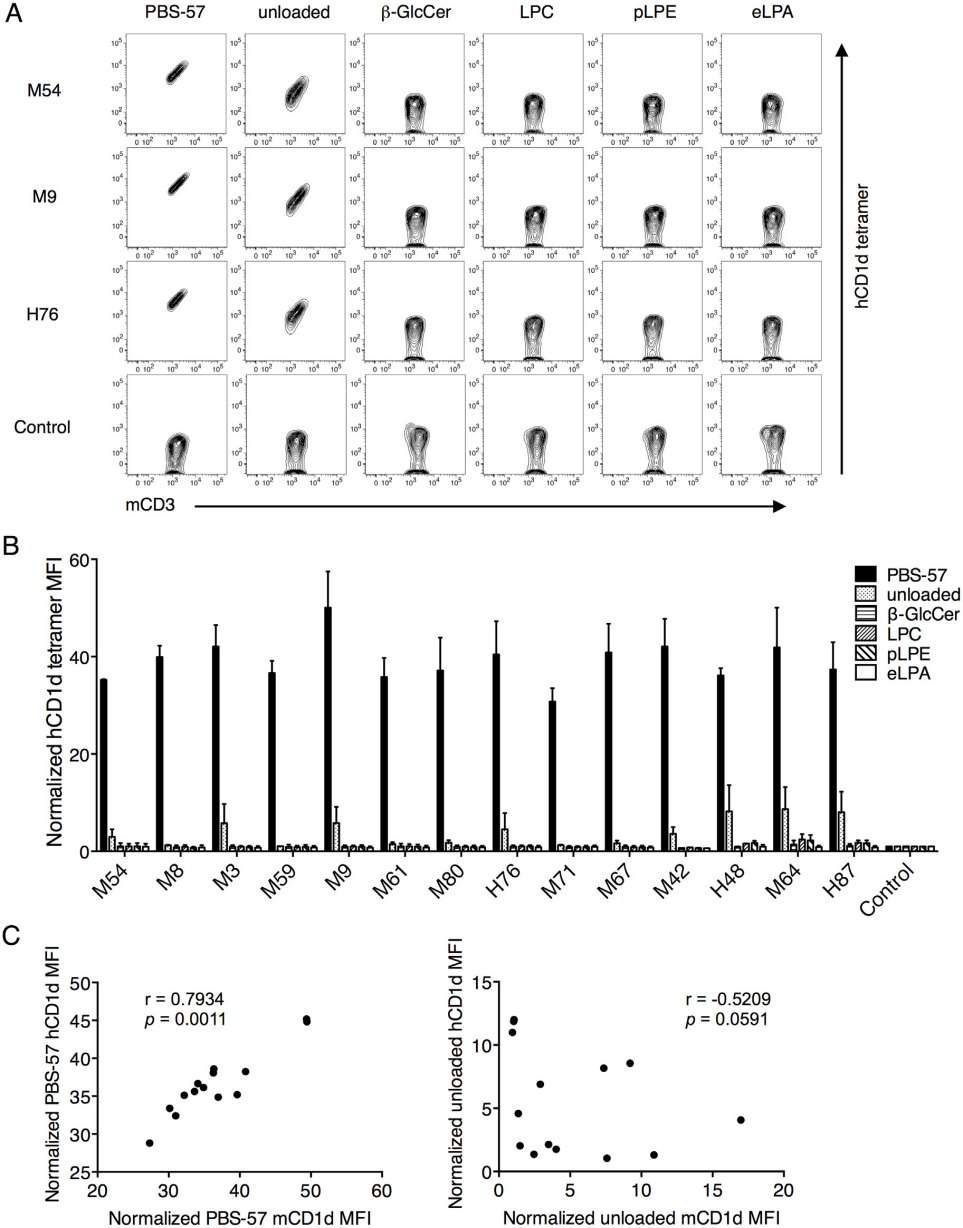
Mouse Vβ8.2 iNKT TCRs recognized only the unloaded hCD1d tetramer but not other self-lipid hCD1d tetramers. (A and B) The same transfectants shown in Fig 3 were stained with anti-mCD3 mAb and unloaded or PBS-57-, β-GlcCer-, LPC-, pLPE-, and eLPA-loaded hCD1d tetramers. MFI values shown are based on CD3^+^ cells. The raw data for three representative clones are shown in A. Staining for all other transfectants are shown in S1 Fig. MFI was normalized to that of control transfectants stained with the same tetramer. Mean values ± SD are shown in the graph. (C) The Spearman correlation coefficients between the MFIs obtained by PBS-57 mCD1d and hCD1d tetramer staining (left), and unloaded mCD1d and hCD1d tetramer staining (right) are shown. The data are representative of two independent experiments.

However, when the same Vβ8.2 TCR transfectants were stained with unloaded, or PBS-57-, β-GlcCer-, LPC-, pLPE-, and eLPA-loaded hCD1d tetramers, we observed that while all of the transfectants were stained by PBS-57 hCD1d tetramer, among the self-antigen tetramers, only the unloaded hCD1d tetramer stained some of the transfectants (Fig 4A, 4B and S1 Fig). Furthermore, the structural reactivity towards the unloaded hCD1d tetramer was not significantly correlated with unloaded mCD1d tetramer. In contrast, a high and significant correlation between the reactivities of mCD1d and hCD1d presenting PBS-57 was observed (Fig 4C). This highlights the unconserved nature of the cross-species iNKT TCR-CD1d-self-lipid interaction, provided that unloaded mCD1d and hCD1d present similar ligands. It is likely since both mCD1d and hCD1d were produced in HEK293 cells.

### Testing Human CD1d Reactivity in a Physiologically Relevant Manner

**Fig 5:**
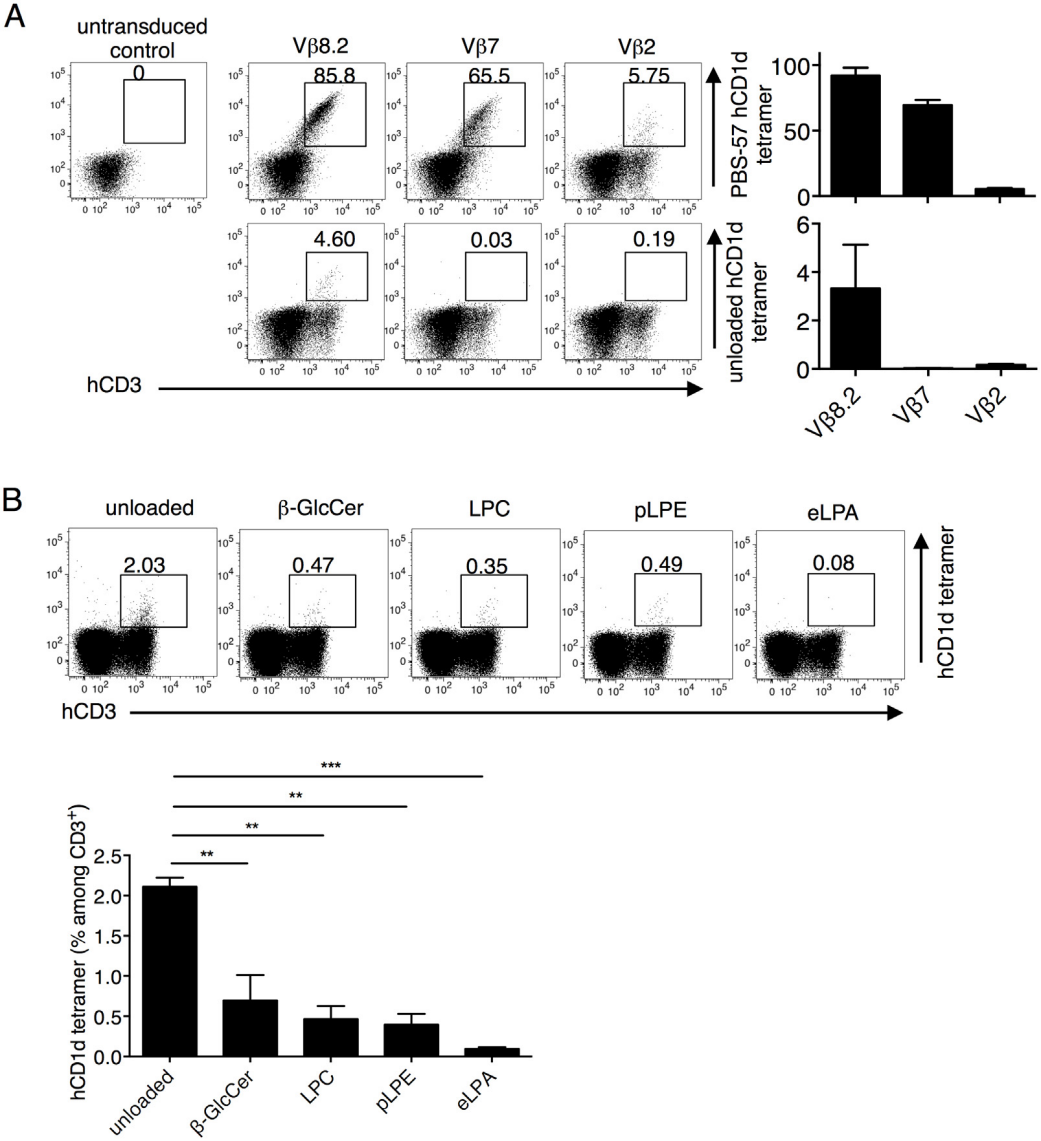
The Jurkat 76.3E1 T cell line as a physiologically relevant host for testing hCD1d reactivity. (A) The unselected Vβ8.2, 7, and 2 libraries were reconstituted in 3E1 cells and stained with anti-human CD3 (hCD3) mAb and unloaded (lower) or PBS-57 loaded (upper) hCD1d tetramers. (B) The unselected Vβ8.2 library transfectants were stained with anti-hCD3 mAb and unloaded, or β-GlcCer-, LPC-, pLPE-, and eLPA-loaded hCD1d tetramers. Self-lipid tetramer staining percentages were compared by one-way ANOVA followed by Bonferroni post-hoc test. ** *p* < 0.01, *** *p* < 0.001.The number above the gate indicates the percentage among the CD3^+^ transfectants. Mean percentages ± SD are shown in the graphs. The data are representative of two to three independent experiments.

Since the only clinically applicable context of the mouse iNKT TCR-hCD1d interaction is that these murine TCRs could be used to redirect the specificity of human T cells, we characterized this interaction in a more physiologically relevant setting. To study murine TCRs in the context of human T cells, we next reconstituted in a human T cell line instead of the mouse 5KC cell line. The Jurkat 76 cell line is a TCR^-/-^ derivative of the human T cell line Jurkat [29]. We transduced Jurkat 76 cells with human CD8αβ and CD4 to mimic the potential roles of co-receptor expression, and then we knocked-down native CD1d expression in the cells with shRNA targeting β2-microglobulin to prevent fraternal activation-induced cell death. After knocking down CD1d, we stably transduced the cells with Vα14i tagged with ΔNGFR and subcloned a cell line, termed Jurkat 76.3E1 (hereafter called 3E1). CD69 expression was upregulated in 3E1 cells upon PMA and ionomycin stimulation (S2 Fig). We reconstituted the unselected mouse iNKT TCRβ libraries in the 3E1 cells, as described above, and stained the transfectants with unloaded or PBS-57 hCD1d tetramer (Fig 5A). Similar results were obtained with the 5KC transfectants. The unselected Vβ8.2 library was further stained with the other self-ligand hCD1d tetramers. Consistent with the clonotypic TCR analysis in Fig 4, the repertoire did preferentially recognize the unloaded hCD1d tetramer but not the other self-ligand loaded hCD1d tetramers (Fig 5B).

### Mouse iNKT TCRs Recognize hCD1d-Lipid Complexes with Greater Ligand Selectivity

**Fig 6:**
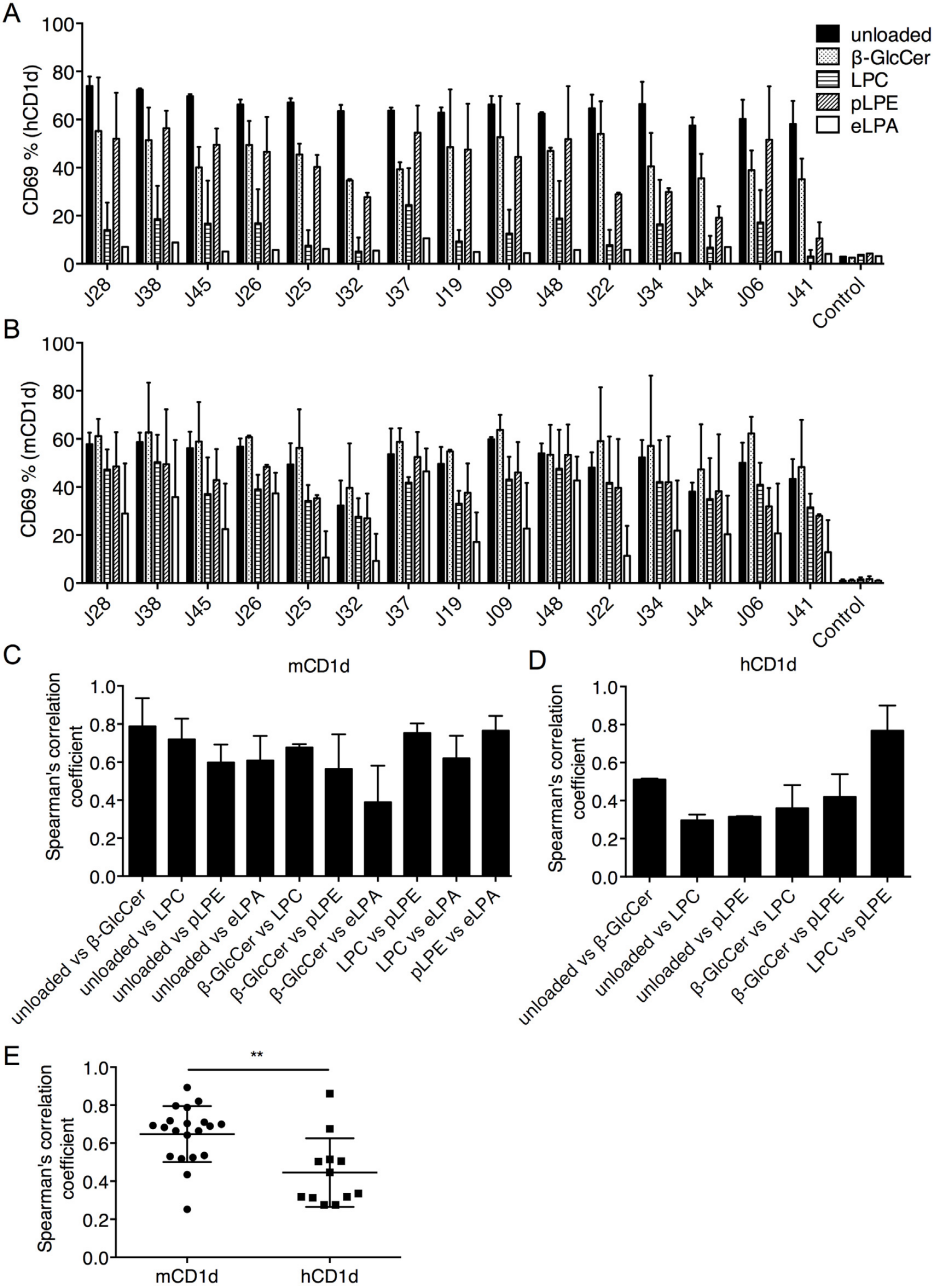
Mouse iNKT TCRs recognize hCD1d-lipid complexes with greater ligand selectivity than mCD1d-lipid complexes. Mouse Vβ8.2 TCRβ chains were cloned from the unloaded hCD1d tetramer-positive population of the unselected library shown in Fig 5B. Clonotypic TCRβ chains were reconstituted in 3E1 cells and stimulated with plate-bound unloaded or β-GlcCer-, LPC-, pLPE-, and eLPA-loaded hCD1d (A) or mCD1d (B) monomers. Surface CD69 expression was measured by flow cytometry after stimulation. The Spearman correlation coefficient values were calculated for the reactivity against the indicated pairs of mCD1d-lipid (C) and hCD1d-lipid (D) complexes. The data are representative of two independent experiments. (E) The Spearman correlation coefficient values pooled from repeated experiments were compared by an unpaired, two-sample t-test. ** *p* < 0.01. Mean values ± SD are shown in the graphs.

The unloaded hCD1d tetramer-positive cells were isolated from 3E1 transfectants expressing the unselected Vβ8.2 library (Fig 5B), as these clones should possess at least some autoreactivity against hCD1d. The TCRβ chains were cloned and individually reconstituted in the 3E1 cells. The sequence information for these TCRs can also be found in S1 Table. Since reactivity against β-GlcCer, LPC, pLPE, and eLPA presented by hCD1d could not be detected by tetramer staining (Fig 5B), we tested antigen reactivity by using a functional assay. The transfectants were stimulated with plate-bound hCD1d or mCD1d, unloaded or loaded with β-GlcCer, LPC, pLPE, or eLPA, and the expression of CD69 was measured (Fig 6A and 6B). The A2/TAX TCR transfectant was used as a negative control. The hCD1d-eLPA complex was not recognized by any of the TCRs and was excluded from further analysis. As expected, the correlation values between the pairs of mCD1d-lipid complexes were moderate to high (Fig 6C). In contrast, the correlation coefficients between the pairs of hCD1d-lipid complexes were significantly lower (Fig 6D and 6E). Collectively, these data demonstrate that, although mouse Vβ8.2 iNKT TCRs do not discriminate among the ligands presented by mCD1d, they recognize hCD1d-lipid complexes with greater ligand selectivity.

## Discussion

In this study, we cloned structurally and/or functionally autoreactive mouse iNKT TCRs and compared the recognition of self-lipids in the context of mouse or human CD1d. Within the three mouse Vβ repertoires, only Vβ8.2 iNKT TCRs possessed detectable staining with hCD1d-self-lipid tetramer. The analysis of clonotypic Vβ8.2 iNKT TCRs demonstrated that although mouse iNKT TCRs recognized different mCD1d-lipid complexes in a conserved manner, they were able to better distinguish the same lipids if presented by hCD1d. Our data further elucidated the difference in cognate and cross-species antigen recognition by murine iNKT TCRs.

The molecular details of iNKT TCRs recognizing cognate CD1d-lipid complexes have been elucidated in depth. Alanine scanning experiments highlighted the necessity of CDR1α, CDR3α, and CDR2β residues in the recognition of CD1d-α-GalCer for both murine and human iNKT TCRs [18, 21, 22, 40, 41]. These findings have been confirmed by crystallographic data, where these CDR loops dominate the TCR footprint on CD1d-α-GalCer [19, 42]. It has also been observed that many of the key residues within CDR1α, CDR3α, and CDR2β loops, critical for CD1d-α-GalCer recognition, are shared between mouse Vβ8.2 and human Vβ11 genes [19, 25, 41]. Therefore, the cross-species recognition mode of murine Vβ8.2 iNKT TCRs for hCD1d-α-GalCer is likely to closely resemble human Vβ11 iNKT TCRs.

In addition to these three CDR loops, CDR3β has been shown play a role in the recognition of cognate CD1d-self-lipid antigens with similar molecular and structural analyses in both species [12, 18, 20, 21, 40, 43]. In particular, the variable CDR3β sequences of autoreactive murine iNKT TCRs tend to interact with Lys148, Val149, and Ala152 residues, which are a part of the α2 helix of mCD1d, regardless of the self-lipid presented [20, 21, 43]. Interestingly, of the three only the Val149 of mCD1d α2 helix is also encoded by hCD1d at the equivalent position [19, 41]. In this study, we observed the staining intensity of unloaded mCD1d and hCD1d tetramers did not correlate (Fig 4C), which strongly suggests that mouse Vβ8.2 CDR3β sequences mediate other interactions beyond the conserved Val on α2 helix of hCD1d presenting self-lipids. More detailed molecular analyses are required to map the residues involved in the interaction between murine CDR3β and hCD1d presenting self-lipids.

Mallevaey et al. demonstrated the contextual importance of CDR1β and CDR2β in permitting CDR3β sequences to confer autoreactivity. Mouse iNKT TCRs encoding random CDR3β library in the presence of wild-type Vβ6 or Vβ8.2 were not stained with the unloaded mCD1d tetramer. However, CDR3β library generated in the context of Vβ6 TCRs with CDR2β derived from Vβ8.2 demonstrated unloaded tetramer positivity [22]. Our data suggest that CDR1β and CDR2β of mouse Vβ8.2 gene is more permissive than those of Vβ7 or Vβ2 in encoding CDR3β sequences that confer cross-species reactivity of hCD1d presenting self-lipids. This was similarly observed for hCD1d-α-GalCer, where not all Vβ7 and Vβ2 murine iNKT TCRs were able to recognize hCD1d presenting the canonical lipid (Fig 2).

At the clonotypic level, we observed that mouse Vβ8.2 iNKT TCRs possessed ligand selectivity when recognizing hCD1d-self-lipid complexes, but the exact underlying molecular mechanism remains elusive. Multiple studies have demonstrated that the hypervariable region of mouse iNKT TCRs, CDR3β, interacts with the monomorphic CD1d molecule and not the ligand [12, 20, 21]. This explains the lack of ligand selectivity for the mCD1d-lipid complexes by distinct clonotypic mouse iNKT TCRs that encode the same Vβ gene. The ligand selectivity between mouse iNKT TCRs with different Vβ usage has been documented [44, 45]. However, in order for the TCRs identified here to distinguish ligands presented by hCD1d, they need to recognize hCD1d-lipid complexes in a manner where the CDR3β region directly interacts with the lipid, or the CDR3β region must indirectly influence the recognition of the CD1d-lipid complex in a sequence-dependent manner. The co-crystallized structures of mouse iNKT TCRs with hCD1d-self-lipids would provide important insights into the molecular basis of this interaction.

Interestingly, we observed that the hCD1d-eLPA complex was not recognized by any of the mouse iNKT TCRs used in this study, in addition to any autoreactive human iNKT TCRs cloned by our group, yet the mCD1d-eLPA complex was recognized by both mouse (Figs 3 and 6) and human iNKT TCRs (unpublished data). To our knowledge, the only lipid identified to date that behaves similarly is iGb3, which can be recognized only when presented by mCD1d, but not hCD1d, by either murine or human iNKT TCRs. In the case of iGb3, the bulky head group of the lipid could not be remodeled when presented by hCD1d to accommodate interactions with iNKT TCRs. Murinizing a key residue of hCD1d that sterically competed with iGb3 restored its stimulatory capacity [46]. Given the major structural differences between eLPA and iGb3, it would be interesting to explore the molecular basis behind the lack of demonstrable antigenicity of eLPA in our experimental system. Nevertheless, we do not rule out the possibility that rare iNKT cell clones could recognize eLPA presented by hCD1d.

Given the recent clinical success of adoptive T cell therapy in cancer, CD1d may be a novel target for overcoming the limitation of HLA restriction and the broadening of the application of this therapy [27]. Currently, HLA-restricted tumor-specific TCRs are transduced into autologous T cells, which are reinfused into the patient. The success of this treatment largely relies on the ability of TCRs to distinguish tumors from healthy tissue. It is known that multiple types of cancer express CD1d [47–49]. Our data present the possibility of using mouse iNKT TCRs to selectively target hCD1d on tumors but not healthy tissue, provided that different lipids are presented in normal and cancerous tissues. Importantly, murine TCRs recognizing HLA-peptide complexes have been successfully used in clinical trials without causing toxic xenoreactivity [50]. Identifying such mouse iNKT TCR(s) could benefit more patients than the targeting of HLA, which is highly polymorphic.

## Conclusion

Using a novel method to generate a mouse iNKT TCR repertoire that included clones with high autoreactivity, we isolated a large panel of clonotypic murine iNKT TCRs and tested their reactivity toward various mCD1d and hCD1d-lipid complexes. Unlike the lack of lipid selectivity within the cognate mouse iNKT TCR-CD1d interactions, mouse Vβ8.2 iNKT TCRs possessed greater ligand selectivity when recognizing hCD1d-lipid complexes. This finding furthers our understanding of the differences between cognate and cross-species reactivity of mouse iNKT TCRs.

## Supporting Information

S1 Fig CD1d tetramer staining of 5KC transfectants. 5KC transfectants shown in Figs 3B and 4B were stained with mouse or human CD1d tetramers, unloaded or loaded with PBS-57, β-GlcCer, LPC, pLPE, or eLPA, and anti-mouse CD3 mAb. Data are representative of two independent experiments. Data is gated on CD3^+^ transfectants. Raw data for M54, M9, and H76 transfectants are shown in Figs 3A and 4A.
(TIFF)

S2 Fig The characterization of Jurkat 76.3E1 cell line. (A) Jurkat 76 and Jurkat 76.3E1 cells were stained with anti-human CD4, CD8α, and CD1d mAbs (black line). The gray solid indicates controls. (B) 3E1 cells stimulated with PMA and ionomycin (black line) and unstimulated (gray solid) were stained with anti-human CD69 mAb. Data are representative of two to three independent experiments.
(TIFF)

S1 Table Sequence information of TCRβ clones used in this study. TCRβ genes from the top section are cloned from 5KC transfectants. TCRβ genes from the bottom section are cloned from Jurkat 76.3E1 transfectants. The usage of Vβ and Jβ genes was defined according to IMGT (http://www.imgt.org/).
(DOCX)
