# Supplementary figures and images for: Mouse and Human CD1d-Self-Lipid Complexes Are Recognized Differently by Murine Invariant Natural Killer T Cell Receptors
Source: PLoS One. 2016 May 23;11(5):e0156114. doi: 10.1371/journal.pone.0156114 (PMC4877060; doi:10.1371/journal.pone.0156114)

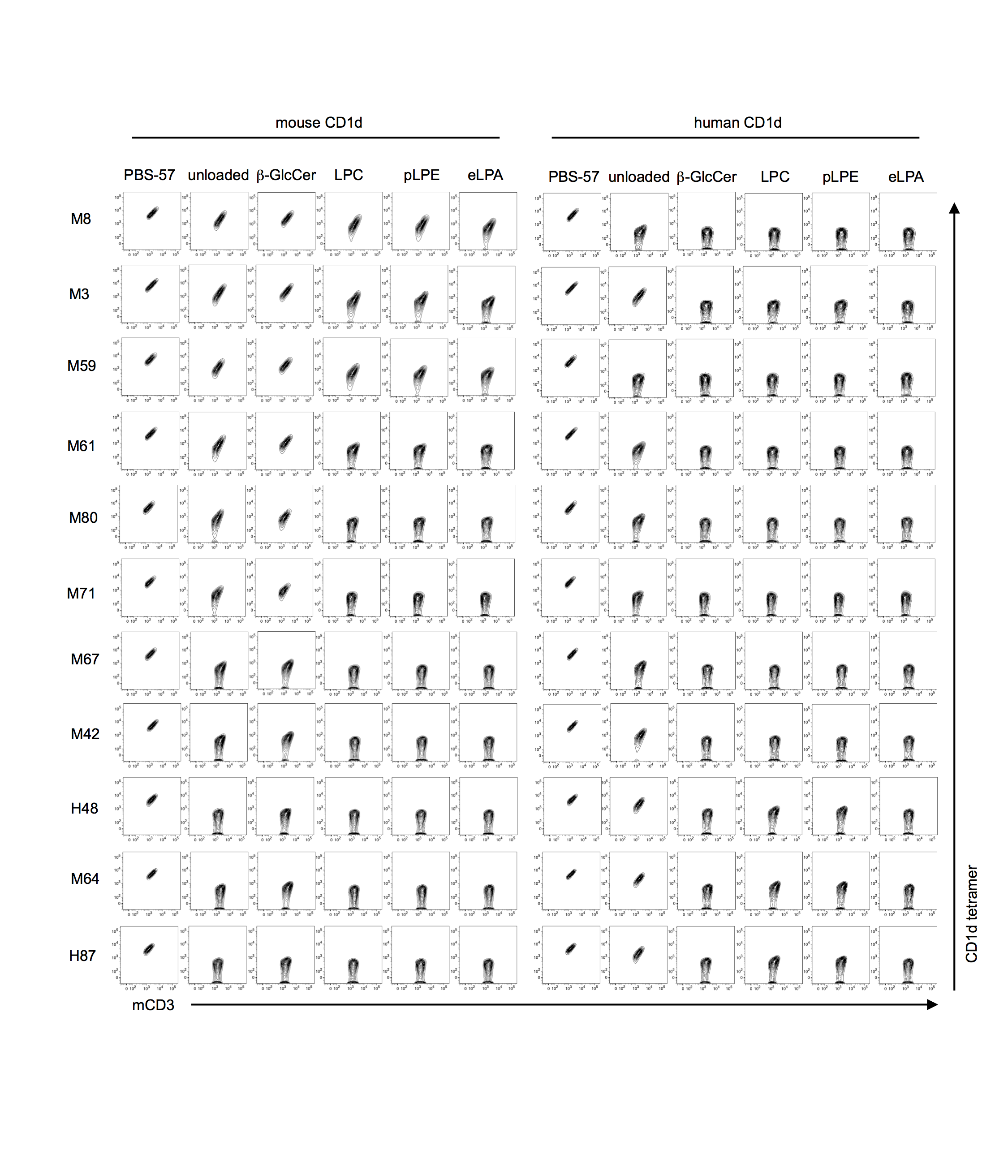

Supplement: S1 Fig — 5KC transfectants shown in Figs 3B and 4B were stained with mouse or human CD1d tetramers, unloaded or loaded with PBS-57, β-GlcCer, LPC, pLPE, or eLPA, and anti-mouse CD3 mAb. Data are representative of two independent experiments. Data is gated on CD3+ transfectants. Raw data for M54, M9, and H76 transfectants are shown in Figs 3A and 4A. (TIFF) [file pone.0156114.s001.tiff]

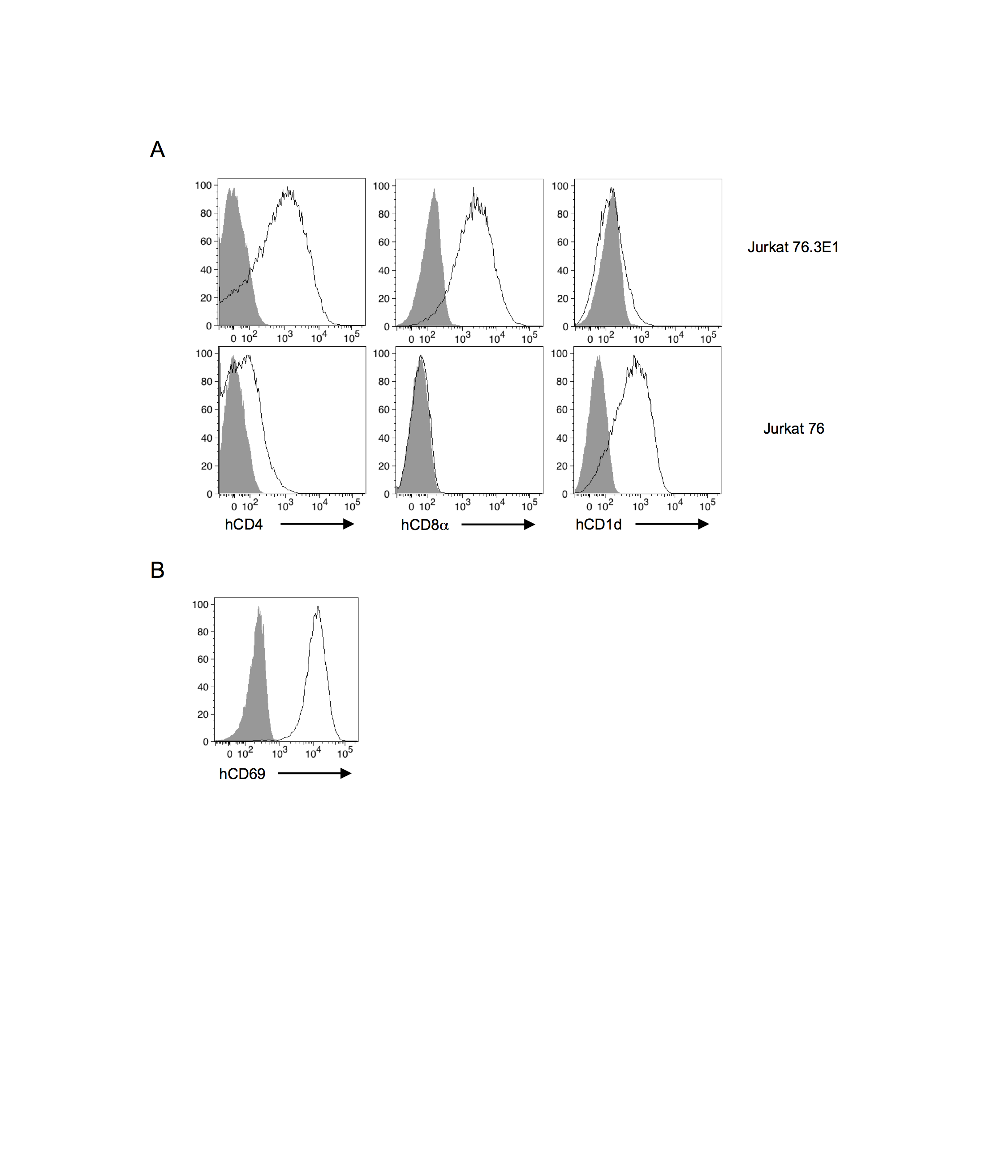

Supplement: S2 Fig — (A) Jurkat 76 and Jurkat 76.3E1 cells were stained with anti-human CD4, CD8α, and CD1d mAbs (black line). The gray solid indicates controls. (B) 3E1 cells stimulated with PMA and ionomycin (black line) and unstimulated (gray solid) were stained with anti-human CD69 mAb. Data are representative of two to three independent experiments. (TIFF) [file pone.0156114.s002.tiff]
