# Supplementary material for: Mouse and Human CD1d-Self-Lipid Complexes Are Recognized Differently by Murine Invariant Natural Killer T Cell Receptors
Source: PLoS One. 2016 May 23;11(5):e0156114. doi: 10.1371/journal.pone.0156114 (PMC4877060; doi:10.1371/journal.pone.0156114)
Supplement: S1 Table — TCRβ genes from the top section are cloned from 5KC transfectants. TCRβ genes from the bottom section are cloned from Jurkat 76.3E1 transfectants. The usage of Vβ and Jβ genes was defined according to IMGT (http://www.imgt.org/). (DOCX) [file pone.0156114.s003.docx]

**Supplementary Table 1. Sequence information of TCRβ clones used in this study**

| Clone | Vβ usage | CDR3β | Jβ gene |
| --- | --- | --- | --- |
| M54 | 8.2 | ASGGLGGPEQYF | 2-7 |
| M08 | 8.2 | ASGDLTGGAEQFF | 2-1 |
| M03 | 8.2 | ASGDWGGTQYF | 2-5 |
| M59 | 8.2 | ASGDWGGAEQFF | 2-1 |
| M09 | 8.2 | ASGDWGGTQYF | 2-7 |
| M61 | 8.2 | ASGDATGGAEQFF | 2-1 |
| M80 | 8.2 | ASGDVGGAGEQYF | 2-7 |
| H76 | 8.2 | ASGGLSYEQYF | 2-7 |
| M71 | 8.2 | ASGDRLGGPEQYF | 2-7 |
| M67 | 8.2 | ASGGTNAEQFF | 2-1 |
| M42 | 8.2 | ASGGQGAEQFF | 2-1 |
| H48 | 8.2 | ASGDAAAEQFF | 2-1 |
| M64 | 8.2 | ASGEGAEQFF | 2-1 |
| H87 | 8.2 | ASGDAAEQYF | 2-7 |
| J06 | 8.2 | ASGDAGEQYF | 2-7 |
| J09 | 8.2 | ASGDDTEQYF | 2-7 |
| J19 | 8.2 | ASGDWTGGPDYTF | 1-2 |
| J22 | 8.2 | ASGDIYEQYF | 2-7 |
| J25 | 8.2 | ATSVGGASDYTF | 1-2 |
| J26 | 8.2 | ASGGLGNYAEQFF | 2-1 |
| J28 | 8.2 | ASGEIEQYF | 2-7 |
| J32 | 8.2 | ASGGEGTNYAEQFF | 2-1 |
| J34 | 8.2 | ASGDAEQFF | 2-1 |
| J37 | 8.2 | ASGDWGAGAEQFF | 2-1 |
| J38 | 8.2 | ASGEGWEQYF | 2-7 |
| J41 | 8.2 | ASGDETGGYEQYF | 2-7 |
| J44 | 8.2 | ASGDRTGGYAEQFF | 2-1 |
| J45 | 8.2 | ASGAPTANSDYTF | 1-2 |
| J48 | 8.2 | ASGDVQGAGEQYF | 2-7 |
